# Analysis of Tumor Mutational Burden, Progression-Free Survival, and Local-Regional Control in Patents with Locally Advanced Non–Small Cell Lung Cancer Treated With Chemoradiation and Durvalumab

**DOI:** 10.1001/jamanetworkopen.2022.49591

**Published:** 2023-01-05

**Authors:** Emily S. Lebow, Annemarie Shepherd, Jordan E. Eichholz, Michael Offin, Daphna Y. Gelblum, Abraham J. Wu, Charles B. Simone, Adam J. Schoenfeld, David R. Jones, Andreas Rimner, Jamie E. Chaft, Nadeem Riaz, Daniel R. Gomez, Narek Shaverdian

**Affiliations:** 1 Department of Radiation Oncology, Memorial Sloan Kettering Cancer Center, New York, New York; 2 Thoracic Oncology Service, Division of Solid Tumor Oncology, Department of Medicine, Memorial Sloan Kettering Cancer Center, New York, New York; 3 Department of Surgery, Memorial Sloan Kettering Cancer Center, New York, New York

## Abstract

**Importance:**

The addition of consolidative durvalumab to chemoradiation has improved disease control and survival in locally advanced non–small cell lung cancer (NSCLC). However, there remains a need to identify biomarkers for response to this therapy to allow for risk adaptation and personalization.

**Objectives:**

To evaluate whether TMB or other variants associated with radiation response are also associated with outcomes following definitive chemoradiation and adjuvant durvalumab among patients with locally advanced unresectable NSCLC.

**Design, Setting, and Participants:**

This cohort study included consecutive patients with unresectable locally advanced NSCLC treated with chemoradiation and adjuvant durvalumab between November 2013 and March 2020 who had prospective comprehensive genomic profiling. This study was completed at a multisite tertiary cancer center. The median (IQR) follow-up time was 26 (21-36) months. Statistical analysis was conducted from April to October 2022.

**Exposures:**

Patients were grouped into TMB-high (≥10 mutations/megabase [mt/Mb]) and TMB-low (<10 mt/Mb) groups and were additionally evaluated by the presence of somatic alterations associated with radiation resistance (*KEAP1*/*NFE2L2*) or radiation sensitivity (DNA damage repair pathway).

**Main Outcomes and Measures:**

The primary outcomes were 24-month local-regional failure (LRF) and progression-free survival (PFS).

**Results:**

In this cohort study of 81 patients (46 [57%] male patients; median [range] age, 67 [45-85] years), 36 patients (44%) had TMB-high tumors (≥10 mt/Mb). Patients with TMB-high vs TMB-low tumors had markedly lower 24-month LRF (9% [95% CI, 0%-46%] vs 51% [95% CI, 36%-71%]; *P* = .001) and improved 24-month PFS (66% [95% CI, 54%-84%] vs 27% [95% CI, 13%-40%]; *P* = .003). The 24-month LRF was 52% (95% CI, 25%-84%) among patients with *KEAP1/NFE2L2*-altered tumors compared with 27% (95% CI, 17%-42%) among patients with *KEAP1/NFE2L2*-wildtype tumors (*P* = .05). On Cox analysis, only TMB status was associated with LRF (hazard ratio [HR], 0.17; 95% CI, 0.03-0.64; *P* = .02) and PFS (HR, 0.45; 95% CI, 0.21-0.90; *P* = .03). Histology, disease stage, Eastern Cooperative Oncology Group status, programmed cell death ligand 1 expression, and pathogenic *KEAP1/NFE2L2*,* KRAS*, and DNA damage repair pathway alterations were not significantly associated with LRF or PFS.

**Conclusions and Relevance:**

In this cohort study, TMB-high status was associated with improved local-regional control and PFS after definitive chemoradiation and adjuvant durvalumab. TMB status may facilitate risk-adaptive radiation strategies in unresectable locally advanced NSCLC.

## Key Points

**Question:**

Is tumor mutational burden (TMB) associated with outcomes among patients with locally advanced unresectable non–small cell lung cancer (NSCLC) treated with definitive chemoradiation and consolidative durvalumab?

**Findings:**

In this cohort study of 81 patients with locally advanced NSCLC treated with definitive chemoradiation and consolidative durvalumab, TMB-high status was associated with significantly improved 24-month locoregional failure and 24-month progression-free survival.

**Meaning:**

These findings suggest that patients with unresectable locally advanced NSCLC with high TMB have a reduced risk of local-regional failure and improved progression-free survival after treatment with definitive chemoradiation and consolidative durvalumab.

## Teaser

This cohort study evaluates whether tumor mutational burden or other variants associated with radiation response are also associated with outcomes following definitive chemoradiation and adjuvant durvalumab among patients with locally advanced unresectable non–small cell lung cancer (NSCLC).

## Introduction

The addition of consolidative durvalumab to concurrent chemoradiation (cCRT) has improved disease control and survival among patients with locally advanced non–small cell lung cancer (NSCLC).^1^ However, there remains a need to identify biomarkers for response to this therapy to allow for risk adaptation and personalization.

In advanced NSCLC, tumor mutational burden (TMB) is a biomarker of immune checkpoint inhibitor (ICI) clinical benefit independent from programmed cell death ligand 1 (PD-L1) expression. Data support increased benefit from ICIs among patients with higher TMB disease^2,3,4,5^ and have resulted in the first US Food and Drug Administration (FDA) histology-agnostic approval of programmed cell death 1 (PD-1) therapy for patients with solid tumors with high TMB (≥10 mutations/megabase [mt/Mb]).^6^ Interestingly, we recently found an association between high TMB and improved local-regional control in patients treated with postoperative radiation therapy without ICI exposure, supporting TMB as a biomarker for radiation sensitivity.^7^ However, TMB has yet to be robustly assessed in patients with locally advanced disease treated with cCRT and durvalumab.^8^

Pathogenic alterations in the KEAP1/NFE2L2 pathway are known to confer resistance to radiation therapy,^9,10,11,12^ although we have previously found that patients treated with cCRT and durvalumab with *KEAP1/NFE2L2*-altered tumors have an attenuated risk of local failure compared with receipt of cCRT alone.^13^ In this study, we sought to reassess this finding with a larger patient cohort and longer follow-up and to increase the sensitivity of this analysis by only comparing patients with functionally significant pathogenic alterations as categorized by OncoKB. OncoKB is the first FDA-recognized tumor mutational database^14^ and contains evidence-based categorizations regarding the functional significance of somatic variants and structural alterations.^15^ We also sought to assess the relative outcomes of pathogenic alterations in the DNA damage repair (DDR) pathways, which are hypothesized to reduce the ability of cells to repair radiation-induced DNA damage, thereby increasing sensitivity to radiation therapy.^16,17^ Furthermore, DDR alterations may increase sensitivity to immune checkpoint inhibition.^18^

In this analysis, we comprehensively assessed TMB and tumor genomic-alterations associated with radiation sensitivity among patients with stage III NSCLC treated with cCRT and durvalumab who had undergone prospective comprehensive genomic testing. To identify genomic biomarkers that could lead to therapy personalization, we compared local-regional disease control and progression-free survival (PFS) among patients with TMB-high tumors vs those with TMB-low tumors and among patients with vs without alterations hypothesized to confer response to radiation therapy.

## Methods

### Study Design

We retrospectively reviewed consecutive patients with American Joint Committee on Cancer eighth edition stage III NSCLC treated between November 2013 and March 2020 who received curative-intent cCRT followed by consolidative durvalumab and underwent comprehensive tissue-based, next-generation sequencing with Memorial Sloan Kettering–Integrated Mutation Profiling of Actionable Cancer (MSK-IMPACT).^19^ Tissue for sequencing was obtained from the primary disease prior to initiation of therapy. All aspects of this study were approved by the institutional review board. The requirement to obtain informed consent was waived for this analysis due to minimal risk to study participants and the impracticality of obtaining consent, and all data were deidentified prior to analysis. This study followed the Strengthening the Reporting of Observational Studies in Epidemiology (STROBE) reporting guideline.^20^

Standard pretreatment evaluation and treatment has been previously described.^13^ Briefly, all patients were evaluated prior to treatment with a physical examination; computed tomography (CT) scan of the chest, abdomen, and pelvis; whole-body fluorine-18 fluorodeoxyglucose positron emission tomography (PET); and magnetic resonance imaging of the head. Patients were treated with curative-intent radiation therapy with standard fractionation (2 Gy per fraction), most often to a total dose 60 Gy (range, 54-66 Gy). Treatment planning included a 4-dimensional CT simulation. Patients were treated with platinum-based doublet chemotherapy concurrent with radiation, followed by consolidative durvalumab (10 mg/kg) every 2 weeks for up to 12 months, as clinically indicated. Imaging with chest CT was standardly performed every 2 to 4 months or more frequently as clinically warranted. All patients suspected of disease progression underwent PET/CT imaging and biopsy when feasible. Patients without biopsy-proven recurrence had overwhelming evidence of recurrence and were treated as such.

### Analysis

**Figure 1.**
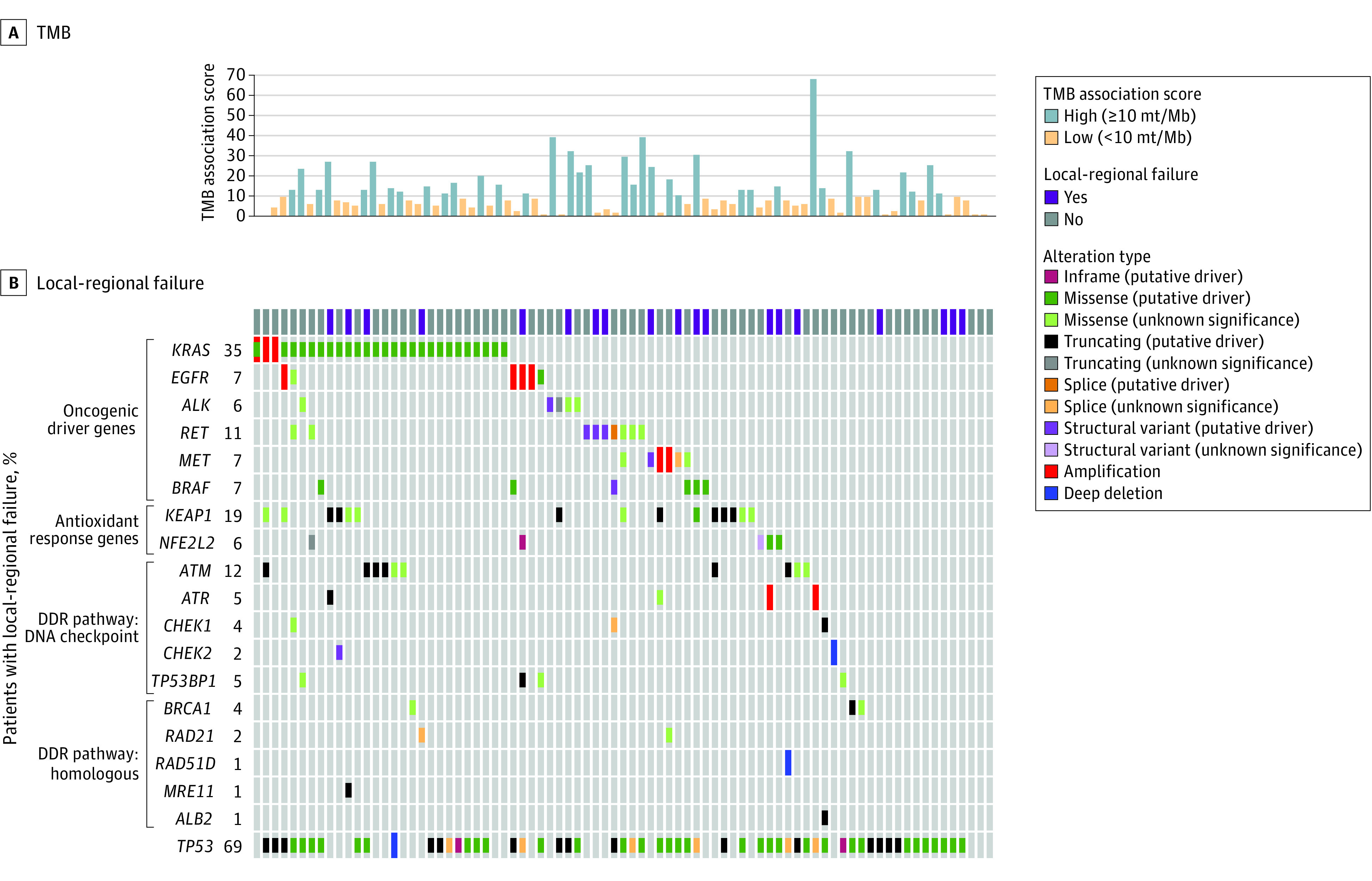
Oncoprint of Selected Alterations Oncoprint of select alterations in National Comprehensive Cancer Center–designated lung cancer driver genes, genes associated with radiation resistance, and genes associated with DNA damage repair (DDR) pathways. Alterations were categorized by OncoKB. Mb indicates megabase, mt, mutation; and TMB, tumor mutational burden.

Age, sex, stage, histology, smoking history, Eastern Cooperative Oncology Group (ECOG) performance status, PD-L1 expression, TMB, alteration status, and time to start of durvalumab from end of radiotherapy were collected. Patients with any self-reported prior smoking history were classified as ever-smokers. Gross tumor volume was obtained from the clinician contours on axial planning CT scans used for delivery of radiotherapy. Tumor alterations were categorized according to OncoKB. Only alterations classified as functionally significant by OncoKB were included in this analysis.^14,15,21,22^ Patients with tumors harboring OncoKB-designated functionally significant alterations (including mutations, amplifications, and structural rearrangements) in radiation resistance genes *KEAP1* or *NFE2L2* were considered as a group, as previously described,^10,11,12,13^ and referred to as having *KEAP1/NFE2L2*-altered disease. Patients with tumors harboring OncoKB-designated functionally significant alterations in listed DDR pathway genes (including DNA checkpoints, Fanconi anemia, and homologous recombination) were also considered as a group^18^ and referred to as having DDR-altered disease (eTable 1 in Supplement 1). Additionally, other genes, including oncogenic driver genes, were assessed as shown in Figure 1.

TMB-high was defined as 10 mt/Mb or greater and TMB-low was defined as fewer than 10 mt/Mb. These categories are consistent with those used in several randomized trials in advanced lung cancer^23,24,25^ and adopted by the FDA for histology-agnostic solid tumor approval of anti–PD-1 therapy.^6^

### Study Outcomes

PFS and local-regional failure (LRF) were defined from the start of radiotherapy until any disease progression or death. Patients were censored at their first progression event.

### Statistical Analysis

Baseline characteristics between patients in the TMB-high and TMB-low groups were compared using the χ^2^ test, Fisher exact, or the Wilcoxon test. The association between patient and tumor characteristics and outcomes including LRF and PFS were evaluated using Cox proportional hazard modeling. PD-L1 was evaluated as a categorical variable (≥1% expression or ≥50% expression). PD-L1 expression was tested per institutional standards with immunohistochemistry using the E13N antibody (Cell Signaling Technology), as previously described.^7^ Immunohistochemistry with PD-L1 E1L3N clone from Cell Signaling was validated against PD-L1 22C3 pharmDx and found to provide highly concordant results for immunohistochemical staining in NSCLC biopsy samples.^26^

Kaplan-Meier analysis was used to estimate overall survival (OS), PFS, and LRF and to compare LRF and PFS outcomes between patients with TMB-high and TMB-low tumors and patients with and without DDR-altered or *KEAP1/NFE2L2*-altered tumors. The log-rank test was used to compare LRF and PFS between groups. Differences were described as statistically significant for *P* ≤ .05, and tests were 2-tailed. All statistical computations were performed using SPSS statistical software version 27 (IBM Corp).

## Results

### Patient and Treatment Characteristics

We identified 81 consecutive patients with stage III NSCLC treated with definitive-intent cCRT and durvalumab, of whom 46 (57%) were male patients and 77 (95%) were prior or current smokers (Table 1). The median (IQR) follow-up was 26 (21-36) months. The median (range) patient age was 67 (45-85) years, 44 patients (54%) had ECOG performance status of 0, and 56 (69%) had adenocarcinoma histology. Most patients had stage IIIB or IIIC disease (57 [71%]). PD-L1 expression was less than 1% among 25 patients (31%), 1% or greater to 49% among 22 patients (27%), 50% or greater among 20 patients (25%), and unavailable among 14 patients (17%).

**Table 1.** Patient Characteristics

Characteristic	Patients, No. (%) (N = 81)
Age, median (range), y	67 (45-85)
Sex	
Female	35 (43)
Male	46 (57)
Smoking history, ever	77 (95)
Performance status, ECOG	
0	44 (54)
1	37 (46)
Histology	
Adenocarcinoma	56 (69)
Squamous cell	18 (22)
Other	7 (9)
PD-L1 expression	
<1%	25 (31)
≥1%-49%	22 (27)
≥50%	20 (25)
Unknown	14 (17)
AJCC eighth edition, overall stage	
IIIA	23 (28)^a^
IIIB	42 (52)
IIIC	15 (19)
T stage	
T1/T0	23 (8)
T2	18 (22)
T3	19 (24)
T4	21 (26)
N stage	
N0	4 (5)
N1	3 (4)
N2	41 (50)
N3	33 (41)
TMB	
Median (IQR), mt/Mb	8.8 (5.3-15.4)
High-TMB	36 (44)
Low-TMB	45 (56)
Pathogenic DDR alteration	
Any	15 (19)
DNA checkpoint^b^	12 (15)
Fanconi anemia	0
Homologous recombination	5 (6)
Pathogenic radiation resistance alteration	
Any^c^	11 (14)
*KEAP1*	8 (10)
*NFE2L2*	3 (4)
Oncogenic driver genes	
*KRAS*	26 (32)
*EGFR*	5 (6)
*ALK*	1 (1)
*RET*	3 (4)
*MET*	4 (5)
*BRAF*	6 (7)
Radiation dose, median (range), Gy	60 (56-66)
Chemotherapy	
Carboplatin/paclitaxel	31 (38)
Carboplatin/pemetrexed	25 (31)
Cisplatin/pemetrexed	18 (22)
Cisplatin/etoposide	6 (7)
Duration of ICI, median (range), mo	6 (2-12)
Early termination of ICI^d^	25 (31)

Abbreviations: AJCC, American Joint Committee on Cancer; DDR, DNA damage repair; ECOG, Eastern Cooperative Oncology Group; ICI, immune checkpoint inhibitor; Mb, megabase; mt, mutation; PD-L1, programmed cell death ligand 1; TMB, tumor mutational burden.

^a^ Sum does not equal to 100% due to rounding.

^b^ Six patients had *ATM *alterations.

^c^ Two patients had co-occurring pathogenic DDR alterations.

^d^ Due to immune-mediated adverse reactions.

Patients were treated with a median of 60 Gy (range, 56-66 Gy) in 2 Gy daily fractions with concurrent platinum-based chemotherapy. Patients were treated with a median (IQR) of 6 (2-12) months of durvalumab that started a median (IQR) of 1.4 months (1.0-2.0) months after completion of cCRT. A total of 25 patients (31%) stopped durvalumab due to toxic effects.

### Genomic Characteristics

Patients underwent prospective, comprehensive genomic profiling with our institutional next-generation targeted sequencing panel (Table 1). The Oncoprint of the cohort contains select alterations of functional significance, including lung cancer driver genes *KEAP1/NFE2L2* and DDR pathway genes (Figure 1). A total of 11 patients (14%) had *KEAP1/NFE2L2*-altered tumors. One or more DDR pathway alterations were detected in 15 patients (19%), including 6 (7%) with pathogenic *ATM* variants. A small group of patients (6 [7%]) had co-occurring *KEAP1/NFE2L2*-altered and DDR-altered tumors.

The median (IQR) TMB was 8.8 (5.3-15.4) mt/Mb, and 36 patients (44%) had TMB-high tumors (>10 mt/Mb) whereas 45 (56%) had TMB-low tumors (eFigure 1 in Supplement 1). Patients with TMB-high and TMB-low tumors were similar in age, sex, smoking history, performance status, PD-L1 status, stage, and both DDR and *KEAP1/NFE2L2* alteration status (Table 2).

**Table 2.** Patient Characteristics Based on TMB

Characteristic	No. (%)		*P* value
	TMB <10 mt/Mb (n = 45)	TMB ≥10 mt/Mb (n = 36)	
Age, median (range), y	66 (45-85)	67 (50-81)	.77
Sex			
Female	16 (36)	19 (53)	.18
Male	29 (64)	17 (47)	
Smoking history, ever	40 (90)	35 (97)	.22
Performance status, ECOG			
0	26 (58)	18 (50)	.51
1	19 (42)	18 (50)	
Histology			
Adenocarcinoma	30 (67)	26 (72)^a^	.76
Squamous cell	11 (24)	7 (19)	
Other	4 (9)	3 (8)	
PD-L1 expression			
<1%	14 (31)	11 (31)	.55
≥1%-49%	11 (24)	22 (27)	
≥50%	14 (31)	11 (31)	
Unknown	6 (13)	8 (22)	
AJCC eighth edition, overall stage			
IIIA	10 (22)	13 (36)	.47
IIIB	25 (56)	17 (47)	
IIIC	9 (20)	6 (17)	
T stage			
T1/T0	12 (27)	11 (30)	.54
T2	11 (24)	7 (19)	
T3	10 (22)	9 (25)	
T4	12 (27)	9 (25)	
N stage			
N0/N1	3 (5)	4 (12)	.61
N2	21 (47)	20 (56)	
N3	21 (47)	12 (33)	
TMB, median (IQR), mt/Mb	6.1 (2.6-7.9)	17.6 (13.2-26.8)	<.001
Pathogenic DDR alterations			
Any	11 (24)	4 (11)	.24
DNA checkpoint^b^	9 (20)	3 (8)	
Fanconi anemia	0	0	
Homologous recombination	4 (9)	1 (3)	
Pathogenic radiation resistance alterations			
Any	7 (16)	4 (11)	.75
*KEAP1*	5 (11)	3 (8)	
*NFE2L2*	2 (4)	1 (3)	

Abbreviations: AJCC, American Joint Committee on Cancer; DDR, DNA damage repair; ECOG, Eastern Cooperative Oncology Group; Mb, megabase; mt, mutation; PD-L1, programmed cell death ligand 1; TMB, tumor mutational burden.

^a^ Sum not equal to 100% due to rounding.

^b^ Overall, 4 patients in the TMB-low group and 2 patients in the TMB-high group had *ATM *alterations.

### Treatment Outcomes

Among all patients, the median OS was not reached. The 12-month and 24-month OS were 93% (95% CI, 87%-99%) and 72% (95% CI, 62%-82%), respectively. A total of 46 patients (45%) had a progression event at median (IQR) of 9 (7-13) months. The median (IQR) PFS was 16 (5-27) months, and 12-month and 24-month PFS were 61% (95% CI, 51%-72%) and 45% (95% CI, 34%-56%), respectively.

In total, 19 patients (23%) had an LRF at a median (IQR) of 10 (7-13) months. The 12-month incidence of LRF was 18% (95% CI, 9%-27%), and 24-month incidence was 31% (95% CI, 1%-43%).

### Association of TMB and Genomic Alterations With LRF

**Figure 2.**
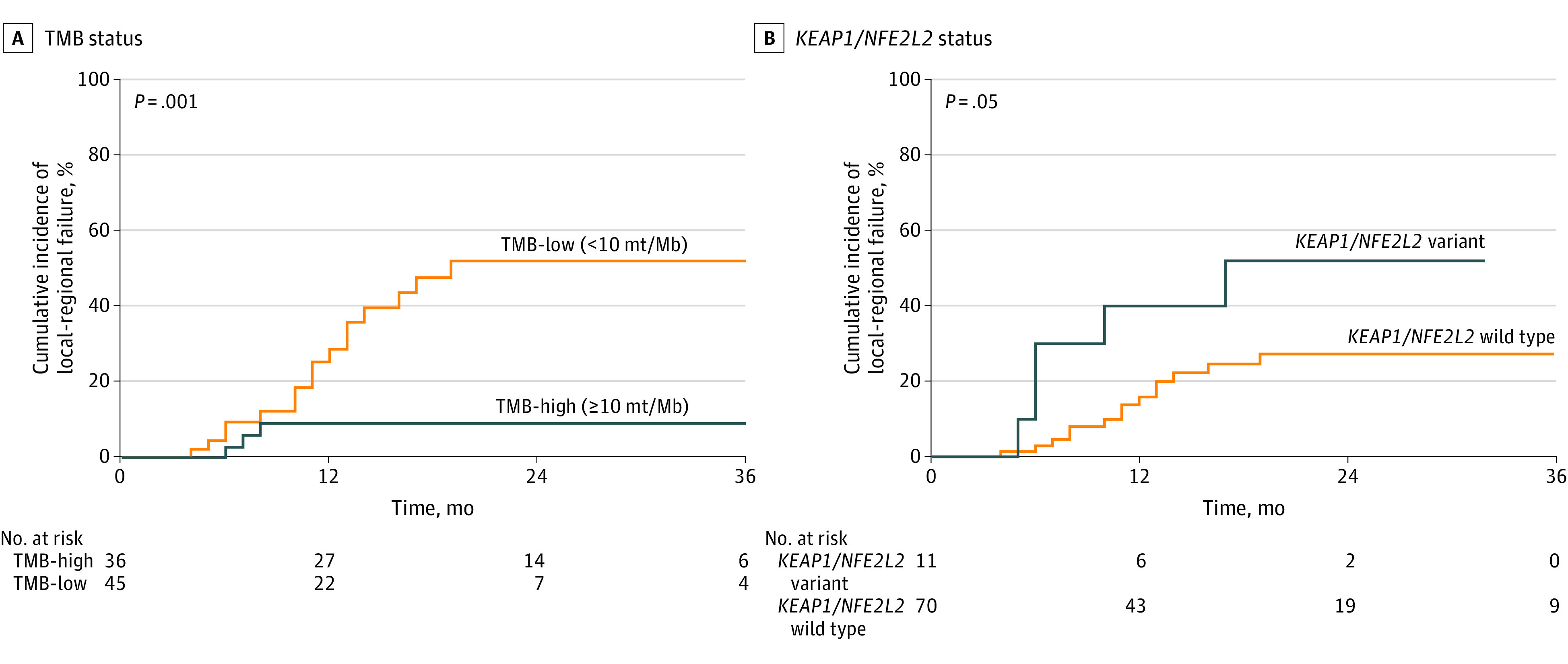
Local-Regional Failure Among Patient Subgroups Mb indicates megabase, mt, mutation; and TMB, tumor mutational burden.

Patients with TMB-high tumors had a significantly lower incidence of LRF compared with patients with TMB-low tumors (Figure 2A). The 24-month cumulative incidence of LRF was 9% (95% CI, 0%-46%) among patients with TMB-high tumors compared with 51% (95% CI, 36%-71%) among patients with TMB-low tumors (*P* = .001). Among 36 patients in the TMB-high group, there were 3 (8%) LRF events occurring at a median of 7 months (range, 6-8 months). Among the 45 patients in the TMB-low group, there were 16 (36%) LRF events at a median (IQR) of 11 (8-13) months.

Patients with *KEAP1/NFE2L2*-altered tumors had an increased risk of LRF. The 24-month LRF was 52% (95% CI, 25%-84%) among patients with *KEAP1/NFE2L2*-altered tumors compared with 27% (95% CI, 17%-42%) among patients with *KEAP1/NFE2L2*-wildtype tumors (*P* = .05) (Figure 2B). Among 11 patients with *KEAP1/NFE2L2*-altered tumors, there were 5 patients (45%) with LRF at a median of 6 (range, 5-17) months. DDR alteration status was not associated with LRF (eFigure 2A in Supplement 1). A total of 4 patients had co-occurring *KEAP1/NFE2L2*-altered and DDR-altered tumors, and among this group 2 patients (50%) had LRF at a 5 and 6 months. Among 14 patients with DDR-altered tumors, there were 5 (36%) LRFs. When patients with isolated DDR-altered tumors were analyzed as a separate subgroup, there was no association with LRF (eFigure 2B in Supplement 1). Among the 6 patients with pathogenic *ATM* alterations, there was 1 LRF failure at 6 months (eFigure 2C in Supplement 1).

Combining TMB and *KEAP1/NFE2L2* variant status identified a patient cohort at very low risk for LRF. Patients with TMB-high tumors without *KEAP1/NFE2L2* alterations had a 7% estimated 24-month incidence of LRF (eFigure 3 in Supplement 1).

On univariate Cox proportional hazard modeling, patients with TMB-high tumors had reduced risk of LRF (hazard ratio [HR], 0.17; 95% 0.03-0.64; *P* = .02). Neither ECOG performance status, histology, stage, PD-L1 expression, DDR, *KEAP1/NFE2L2*,* KRAS*, or* TP53* variant status was associated with LRF (eTable 2 in Supplement 1). Additionally, when analyzed as a continuous variable, a higher TMB remained significantly associated with a reduced risk of LRF (HR, 0.89; 95% CI, 0.80-0.97, *P* = .02).

### Association of TMB and Genomic Alterations With PFS

**Figure 3.**
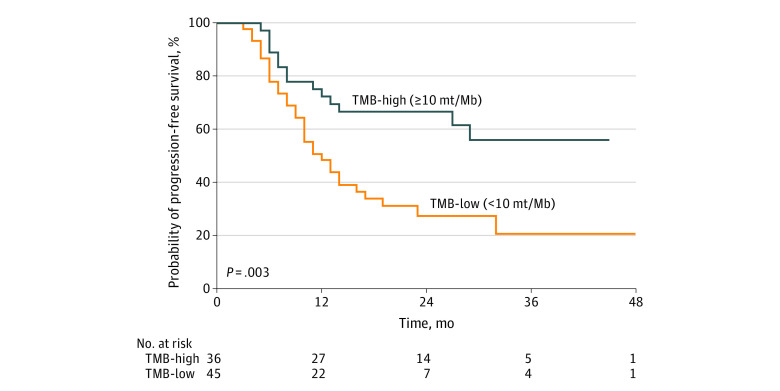
Comparison of Progression-Free Survival Comparison of progression-free survival among patients with tumor mutational burden (TMB)–high (>10 mutations/Megabase [mt/Mb]) vs TMB-low (<10 mt/Mb) tumors.

Compared with patients with TMB-low tumors, patients with TMB-high tumors had improved PFS: the 24-month PFS among patients with TMB-high tumors was 66% (95% CI, 54%-84%) compared with 27% (95% CI, 13%-40%) among patients with TMB-low tumors (HR, 0.40; 95% CI, 0.23-0.72; *P* = .003) (Figure 3). However, patients with and without KEAP*1/NFE2L2*-altered tumors had similar PFS (eFigure 4 in Supplement 1). On univariate Cox proportional hazard modeling, patients with TMB-high tumors had improved PFS (HR, 0.45; 95% CI, 0.21-0.90; *P* = .03), while other tumor and patient factors, including DDR and *KEAP1/NFE2L2* alteration status, were not significantly associated with PFS (eTable 3 in Supplement 1).

## Discussion

In this study, we presented a large cohort of patients with stage III NSCLC treated with cCRT and durvalumab who underwent prospective comprehensive genomic testing. With a median follow-up of more than 2 years following cCRT and consolidative durvalumab, we found that patients with TMB-high tumors had more than a 5-fold reduction in the risk of LRF compared with patients with TMB-low tumors. Furthermore, TMB-high status was associated with improved PFS. Additionally, in this analysis limited to functionally significant variants by OnkoKB, we found *KEAP1/NFE2L2*-altered tumors to have an increased risk of LRF. These data provide the groundwork to appropriately select patients with unresectable stage III NSCLC for risk-adaptative strategies based on tumor genomics.

There are several mechanisms by which TMB may affect response to multimodal therapy in the setting of locally advanced NSCLC. We have previously found that TMB-high status was associated with reduced risk of LRF following postoperative radiation therapy in patients with lung cancer without ICI exposure, suggesting that TMB status may serve as a potential novel biomarker for radiation sensitivity.^7^ High TMB levels have been associated with alterations in DDR genes and, given their role in radiation repair, a finding of improved LRF outcomes in TMB-high tumors would be logical.^27,28^

It is also possible that TMB-high tumors have improved response to immune checkpoint inhibition, as several studies have found an association between TMB and clinical benefit from ICIs.^4,23,29^ TMB may serve as a surrogate for genomic instability and for tumor neoantigen presentation, making TMB-high tumors more immunogenic and, therefore, more likely to respond to immune checkpoint inhibition.^30^ Additionally, data have found radiation sensitivity to also be partly dependent on the antitumor immune response, therefore providing further biological rationale for increased radiation sensitivity in patients with TMB-high tumors.^31,32^

TMB may have potential as a complementary biomarker to PD-L1 expression in patients with locally advanced unresectable NSCLC. In the PACIFIC trial, both patients who had PD-L1 expression of less than 25% and 25% or greater derived a PFS and OS benefit from durvalumab following chemoradiation.^1^ While a more recent analysis from the PACIFIC-R study^33^ found improved disease control in patients with PD-L1 expression of 1% or greater vs less than 1%, other multicenter studies have not demonstrated associations of PD-L1–negative and PD-L1–low status with disease control.^34^ TMB has been found to be a biomarker for sensitivity to PD-1/PD-L1 inhibition across PD-L1 expression subgroups,^35^ highlighting the potential for TMB to serve as a biomarker independent of PD-L1 expression levels in the setting of locally advanced NSCLC.

While evidence suggests TMB is associated with response to therapy, the prognostic association between TMB and outcomes in NSCLC independent of therapy has been equivocal. In a study of resected early stage lung cancer, high TMB was associated with more aggressive pathologic features, including lymphovascular invasion and spread through airway space, but was not independently prognostic for survival.^36^ A recent pancancer analysis did not find higher TMB to be associated with better prognosis in patients not treated with ICIs and specifically found poor survival in patients with NSCLC and higher TMB not treated with ICIs.^37^ Other studies have reported both high TMB and low TMB to be associated with improved outcomes in resected lung cancer.^38,39^ Similarly, while KEAP1/NFE2L2 pathway alterations are associated refractoriness to radiation and systemic therapy, their role as independent prognostic factors in NSCLC is equivocal.^40^

These data require validation but suggest that TMB can be used as an integral biomarker to selectively risk-adapt thoracic radiotherapy with both intensification and deintensification strategies. Despite the genomic complexity of lung cancer, all patients with inoperable locally advanced lung cancer are generally treated with a similar thoracic radiotherapy dose of approximately 60 Gy.^41^ Uniform dose escalation to all patients does not offer clinical benefit in part due to increased toxic effects at higher radiotherapy doses.^42,43^ While the addition of consolidative durvalumab improves outcomes, including a reduction in LRF,^13,44,45^ we still observed a 31% risk of LRF at 24 months. Given the importance of local-regional disease control on survival in lung cancer,^46^ further efforts are warranted to improve outcomes, and low-TMB status can potentially select patients for treatment-intensification strategies.

Equally important, deintensification strategies warrant investigation to improve the therapeutic ratio of thoracic radiation in selected patients. Of note, 31% of patients experienced toxic effects from durvalumab requiring early termination, and there are many known toxic effects of ICI, highlighting the importance of appropriately selecting patients most likely to benefit from immune checkpoint inhibition. By selecting patients based on both TMB and *KEAP1/NFE2L2* variant status, we identified a patient cohort at very low risk of local-regional recurrence. These patients may also benefit from risk-adaptive strategies. Prior studies in the setting of postoperative radiation therapy in NSCLC have found even small, 5 to 10 Gy, reductions in the radiation dose to the mediastinum to result in improved survival and less intercurrent disease.^47,48^ Therefore, by fine-tuning radiation dose, with even a modest dose reduction, we could improve the therapeutic ratio and potentially survival outcomes in patients with treatment-sensitive disease.

Incorporation of TMB-high status as a biomarker for lung cancer has been complicated by use of various cutoffs in major clinical trials^2,49,50^ and by variation in technical approaches to TMB measurement, including whole-exome sequencing vs targeted panel sequencing approaches.^51^ In this cohort, TMB was determined by an FDA-authorized next-generation assay, MSK-IMPACT, which sequences targeted panels of cancer-related genes using tumor-derived and matched normal germline DNA. It will be essential to harmonize TMB measurement across diagnostic platforms^52^ for reliable utilization as a biomarker in lung cancer. Nonetheless, TMB likely has a role as a biomarker in advanced lung cancer.

Prior studies in patients treated with cCRT alone have found *KEAP1/NFE2L2-*altered tumors to be at significantly higher risk for LRF.^9,10,11^ Although we previously did not find a strong association between *KEAP1/NFE2L2*-altered status and local-regional control in patients treated with cCRT and durvalumab,^13^ our and other prior analyses had limitations. In the present study, the functional significance of all genomics alterations were categorized according to OncoKB, and only functionally significant pathogenic alterations were considered in the analyses.

Through this more sensitive analysis in a larger patient cohort, we observed that patients with pathogenic *KEAP1/NFE2L2*-altered tumors had an increased risk of LRF. Prior data have found that *KEAP1/NFE2L2* alterations are not just binary activators of the NRF2 pathway but rather have a continuous range of associations with radiation sensitivity across alterations.^53^ Our current finding that selected variants with known pathological function have greater associations with outcomes suggests that the addition of durvalumab may be sufficient for certain alterations but potentially not those that more strongly activate the NRF2 pathway. Additionally, we did not find *KEAP1/NFE2L2* alterations to be associated with poor PFS, suggesting that while these alterations can be associated with radiotherapy outcomes, they may not be as consequential to ICI outcomes.

Interestingly, we did not find pathogenic alterations in DDR genes to be associated with local-regional outcomes. As the DDR pathway plays a role in radiation repair, pathogenic alterations would be hypothesized to lead to radiation sensitivity. Prior studies that have found patients with DDR pathway gene alterations to have improved local-regional outcomes after radiotherapy were mostly limited to patients without subsequent ICI exposure.^7,54^ This suggests that the local-regional control benefit imparted by consolidative ICI treatment could minimize the difference in outcomes between patients with and without alterations in DDR genes. Additionally, we assessed a large panel DDR genes that would be anticipated to have variable effects on radiation response.^55,56^ Although we found alterations in *ATM* to be most common, and multiple lines of evidence have found *ATM* alterations to impart radiation sensitivity,^17^ our analysis may have been underpowered to detect a clinically meaningful difference.

### Limitations

This study has limitations, including its retrospective design at a single tertiary academic medical center and small size of genetic subgroups, which may limit the analyses. Tumor genomic profiling was completed prospectively at the discretion of treating clinicians, and we included all consecutive patients with genomic profiling, including patients with *EGFR* driver alterations who are less likely to derive benefit from the PACIFIC regimen. While alterations in the DDR pathway and *KEAP1/NFE2L2* were not functionally validated, we did use OncoKB, an FDA-recognized tumor mutational database, to categorize alterations identified by tissue-based sequencing in this cohort.^15^ To maintain consistency with the field, we primarily used a binary cutoff to evaluate TMB-high vs TMB-low cohorts. This cutoff has been used as the basis of FDA-approval for anti–PD-1 therapy given data supporting its utility as a biomarker.^6^ However, we additionally assessed TMB as a continuous variable to support our findings.

## Conclusions

In this cohort study of 81 patients with stage III NSCLC who underwent prospective comprehensive genomic testing and were treated with cCRT and durvalumab, we found that patients with TMB-high tumors had significantly reduced risk of LRF and improved PFS. TMB is a promising biomarker for guiding trials of personalized therapy approaches, including intensification of therapy for high-risk patients and deintensification of therapy for low-risk patients with stage III NSCLC.

## Notes

Supplement 1. **eFigure 1.** Distribution of TMB Among Patients in This Study **eFigure 2.** Comparison of Outcomes Among Patients With and Without DDR-Altered Tumors **eFigure 3.** Comparison of Cumulative Incidence of Local-Regional Failure Among Patients With TMB-High (>10 mt/Mb) and KEAP1/NFE2L2-Wildtype vs All Other Patients **eFigure 4.** Comparison of Progression-Free Survival Probability Among Patients With and Without KEAP1/NFE2L2 Alterations **eTable 1.** Investigated Genes Associated With DNA Damage Response and Repair (DDR) by Specific DDR Pathways and Incidence of Pathogenic Alterations **eTable 2.** Factors Associated With Local-Regional Failure **eTable 3.** Factors Associated With Progression-Free Survival

Supplement 2.
Data Sharing Statement
