# Supplementary material for: Analysis of Tumor Mutational Burden, Progression-Free Survival, and Local-Regional Control in Patents with Locally Advanced Non–Small Cell Lung Cancer Treated With Chemoradiation and Durvalumab
Source: JAMA Netw Open. 2023 Jan 5;6(1):e2249591. doi: 10.1001/jamanetworkopen.2022.49591 (PMC9856786; doi:10.1001/jamanetworkopen.2022.49591)
Supplement: Supplement 1. — eFigure 1. Distribution of TMB Among Patients in This Study eFigure 2. Comparison of Outcomes Among Patients With and Without DDR-Altered Tumors eFigure 3. Comparison of Cumulative Incidence of Local-Regional Failure Among Patients With TMB-High (>10 mt/Mb) and KEAP1/NFE2L2-Wildtype vs All Other Patients eFigure 4. Comparison of Progression-Free Survival Probability Among Patients With and Without KEAP1/NFE2L2 Alterations eTable 1. Investigated Genes Associated With DNA Damage Response and Repair (DDR) by Specific DDR Pathways and Incidence of Pathogenic Alterations eTable 2. Factors Associated With Local-Regional Failure eTable 3. Factors Associated With Progression-Free Survival [file jamanetwopen-e2249591-s001.pdf]

Supplemental Online Content

Lebow ES, Shepherd A, Eichholz JE, et al. Analysis of tumor mutational burden, progression-free survival, and local-regional control in patents with locally advanced non–small cell lung cancer treated with chemoradiation and durvalumab. *JAMA Netw Open.* 2023;6(1):e2249591. doi:10.1001/jamanetworkopen.2022.49591

eFigure 1. Distribution of TMB Among Patients in This Study

eFigure 2. Comparison of Outcomes Among Patients With and Without DDR-Altered Tumors

eFigure 3. Comparison of Cumulative Incidence of Local-Regional Failure Among Patients With TMB-High (>10 mt/Mb) and KEAP1/NFE2L2-Wildtype vs All Other Patients

eFigure 4. Comparison of Progression-Free Survival Probability Among Patients With and Without KEAP1/NFE2L2 Alterations

eTable 1. Investigated Genes Associated With DNA Damage Response and Repair (DDR) by Specific DDR Pathways and Incidence of Pathogenic Alterations

eTable 2. Factors Associated With Local-Regional Failure

eTable 3. Factors Associated With Progression-Free Survival

This supplemental material has been provided by the authors to give readers additional information about their work.

© 2023 Lebow ES et al. *JAMA Network Open.*

eFigure 1. Distribution of TMB Among Patients in this Study

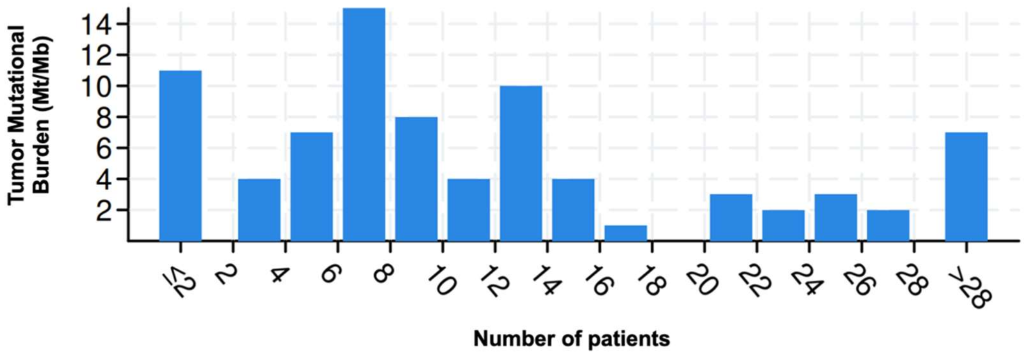

Distribution of tumor mutational burden (TMB) among patients included in this study as determined by tissue-based sequencing of primary disease by MSK-IMPACT. TMB of > 10 mutations per megabase (Mt/Mb) was categorized as TMB-high, and TMB < 10 Mt/Mb was categorized as TMB-low.

© 2023 Lebow ES et al. *JAMA Network Open.*

eFigure 2. Comparison of Outcomes Among Patients With and Without DDR-Altered Tumors.

A.

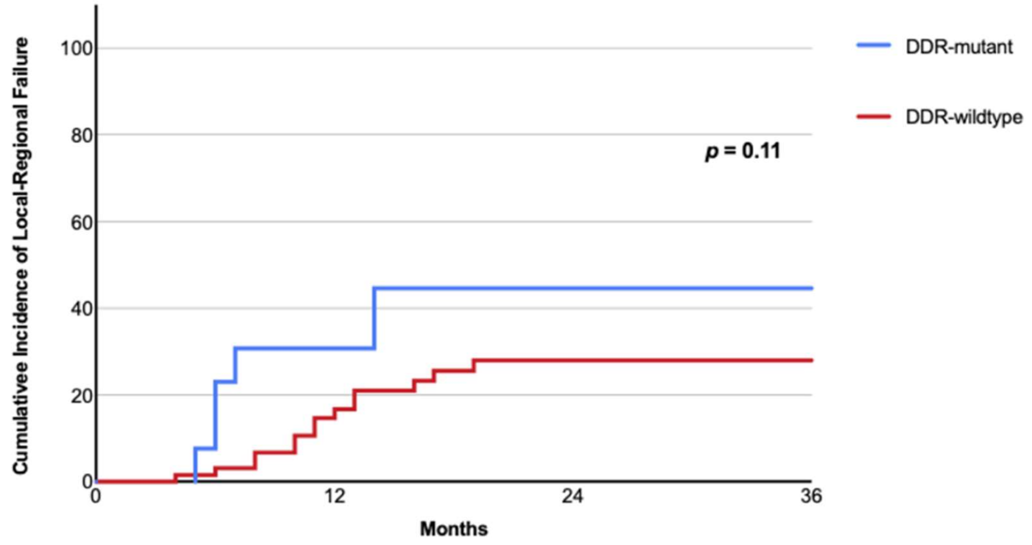

DDR-mutant	14	8	2	2
DDR-wildtype	67	41	20	8

B.

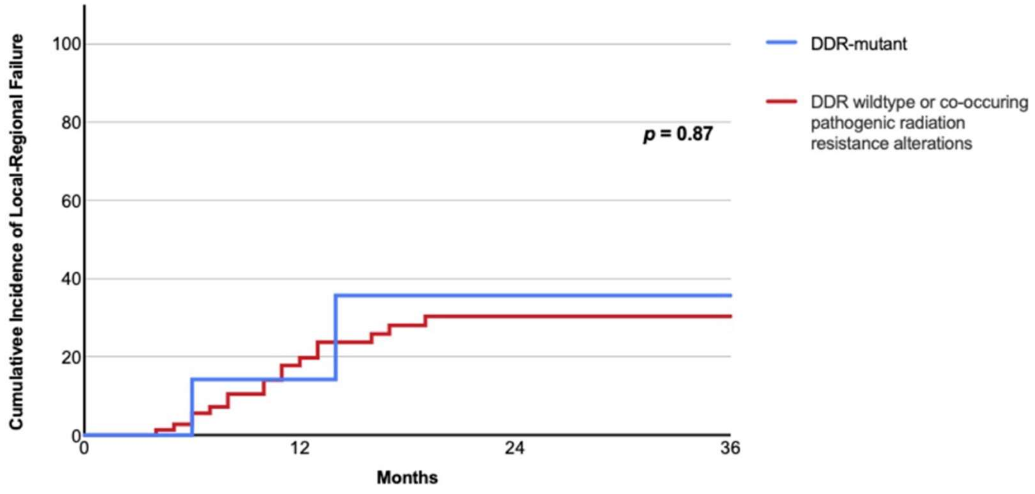

DDR-mutant	7	6	2	2
DDR-wildtype or co-occurring KEAP1/NFE2L2-mutant	74	43	20	8

© 2023 Lebow ES et al. *JAMA Network Open.*

C.

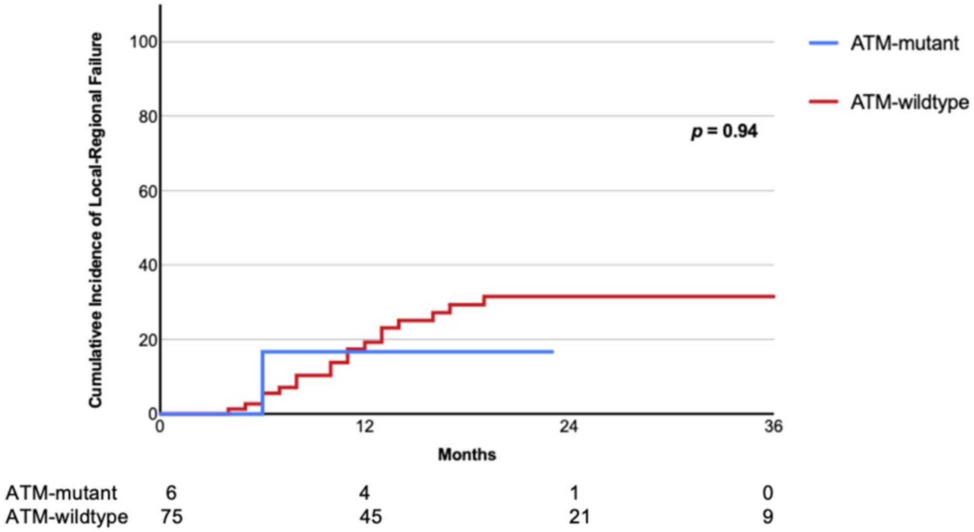

A) Cumulative incidence of local failure local- regional failure. B) Cumulative incidence of local-regional failure among patients with and isolated DDR-mutant tumors to patients with DDR-wildtype tumors or co-occurring KEAP1/NFE2L2-mutant tumors. C) Comparison of cumulative incidence of local failure local-regional between patients with ATM-mutant and ATM-wildtype tumors. DDR: DNA Damage Repair; ATM: Ataxia telangiectasia, mutated

© 2023 Lebow ES et al. *JAMA Network Open.*

eFigure 3. Comparison of Cumulative Incidence of Local-Regional Failure Among Patients With TMB-High (>10 mt/Mb) and KEAP1/NFE2L2-wildtype vs All Other Patients

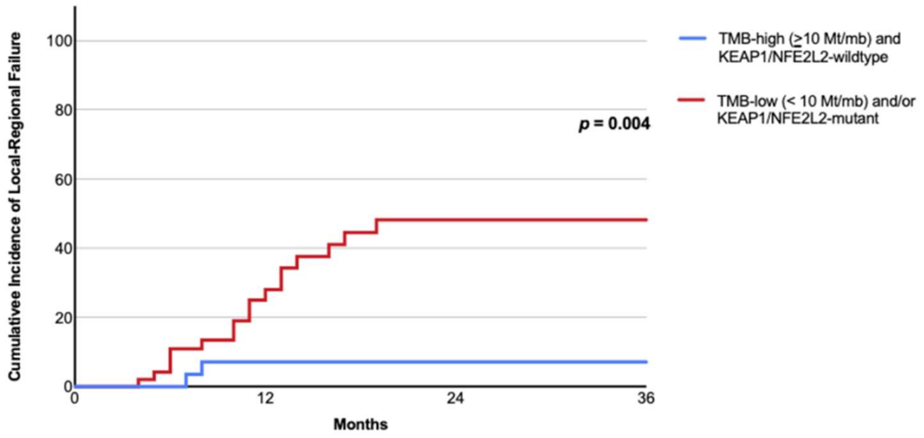

TMB-high and KEAP1/NFE2L2-wildtype	32	24	12	6
TMB-low and/or KEAP1/NFE2L2-mutant	49	25	9	5

TMB: Tumor mutational burden; Mt: Mutations; Mb: Megabase

© 2023 Lebow ES et al. *JAMA Network Open.*

eFigure 4. Comparison of Progression-Free Survival Probability Among Patients With and Without KEAP1/NFE2L2 Alterations

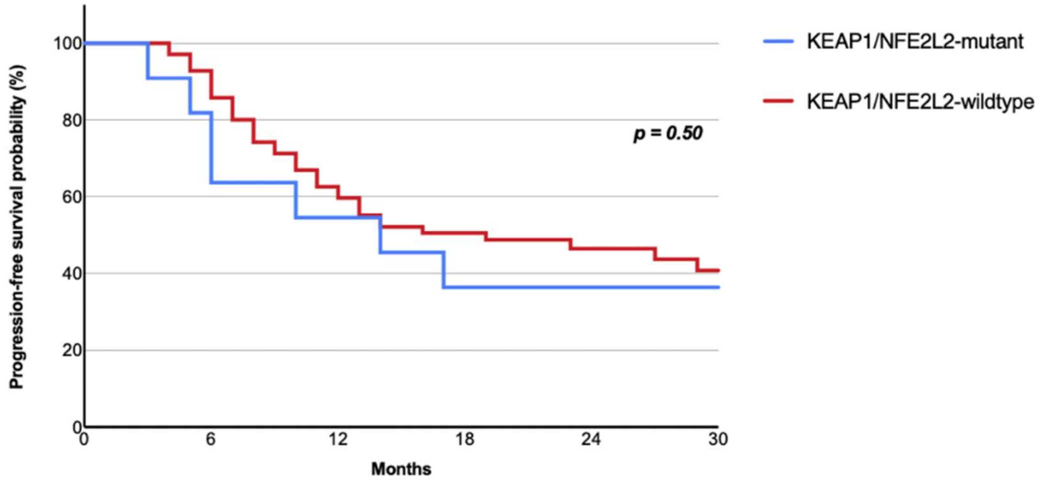

KEAP1/NFE2L2-mutant	11	9	7	5	3	2
KEAP1/NFE2L2-wildtype	70	65	43	30	19	14

© 2023 Lebow ES et al. *JAMA Network Open.*

eTable 1. Investigated Genes Associated With DNA Damage Response and Repair (DDR) by Specific DDR Pathways and Incidence of Pathogenic Alterations

DNA Checkpoints (Fork QC)	Fanconi Anemia (FA) repair	Homologous Recombination (HR)
ATM	FANCA	BARD1
ATR	FANCC	BLM
CHEK1		BRCA1
CHEK2		BRCA2
MDC1		BRIP1
TP53BP1		MRE11
		NBN
		PALB2
		RAD21
		RAD50
		RAD51
		RAD51B
		Rad51C
		RAD51D
		RAD52
		RAD54L
		SLX4
		XRCC2

QC, quality control

© 2023 Lebow ES et al. *JAMA Network Open.*

eTable 2. Factors Associated With Local-Regional Failure

Variable	Univariate	
	HR (95% CI)	p Value
ECOG 0 vs ECOG ≥ 1	1.21 (0.41-3.39)	0.713
Adenocarcinoma vs squamous cell histology	0.55 (0.20 - 1.58)	0.255
Stage IIIC vs Stage IIIA/IIIB	1.43 (0.38 - 4.90)	0.578
PD-L1 < 1%	0.37 (0.05 - 2.86)	0.326
PD-L1 1 – 49%	0.95 (0.16 - 6.0)	0.956
PD-L1 > 50%	0.24 (0.03 - 1.73)	0.152
TMB high (≥10 Mt/mb) vs TMB low (<10 Mt/mb)	0.17 (0.03 - 0.64)	**0.022**
DDR-mutant	1.23 (0.31 - 4.14)	0.748
KEAP1/NFE2L2-mutant	1.64 (0.37 - 5.30)	0.453
KRAS-mutant	0.40 (0.18 – 1.72)	0.402
TP53-mutant	0.55 (0.22 – 1.49)	0.214
T3/4 vs T1/T2	1.49 (0.60 – 3.87)	0.386
N3 vs N1/N2	1.59 (0.64 – 4.00)	0.316
Gross tumor volume > median	1.79 (0.66 – 5.26)	0.262

TMB: Tumor mutational burden; Mt: mutation; Mg: megabase; Gy: Gray; DDR: DNA Damage Repair

© 2023 Lebow ES et al. *JAMA Network Open.*

eTable 3. Factors Associated With Progression-Free Survival

Variable	Univariate	
	**HR (95% CI)**	**p Value**
ECOG 0 vs ECOG ≥ 1	1.43 (0.74 – 2.73)	0.283
Adenocarcinoma vs squamous cell histology	0.80 (0.42 – 1.61)	0.522
Stage IIIC vs Stage IIIA/IIIB	0.89 (0.36 – 2.0)	0.779
PD-L1 < 1%	1.99 (0.60 - 7.92)	0.284
PD-L1 1 – 49%	2.09 (0.62 - 8.40)	0.258
PD-L1 > 50%	0.87 (0.23 - 3.65)	0.842
TMB high (≥10 Mt/mb) vs TMB low (<10 Mt/mb)	0.45 (0.21 – 0.90)	**0.030**
DDR-mutant	1.28 (0.54 – 2.78)	0.545
KEAP1/NFE2L2-mutant	1.27 (0.42 – 3.04)	0.629
T3/4 vs T1/T2	1.15 (0.64 –2.0)	0.634
N3 vs N1/N2	1.13 (0.62 – 2.03)	0.684
Gross tumor volume > median	1.45 (0.79 – 2.74)	0.235

TMB: Tumor mutational burden; Mt: mutation; Mg: megabase; Gy: Gray; DDR: DNA Damage Repair

© 2023 Lebow ES et al. *JAMA Network Open.*
