# Supplementary material for: Analysis of Tumor Mutational Burden, Progression-Free Survival, and Local-Regional Control in Patents with Locally Advanced Non–Small Cell Lung Cancer Treated With Chemoradiation and Durvalumab
Source: JAMA Netw Open. 2023 Jan 5;6(1):e2249591. doi: 10.1001/jamanetworkopen.2022.49591 (PMC9856786; doi:10.1001/jamanetworkopen.2022.49591)
Supplement: Supplement 2. — Data Sharing Statement [file jamanetwopen-e2249591-s002.pdf]

Data Sharing Statement

Lebow. Analysis of Tumor Mutational Burden, Progression-Free Survival, and Local-Regional Control in Patents with Locally Advanced Non-Small Cell Lung Cancer Treated With Chemoradiation and Durvalumab. *JAMA Netw Open*. Published January 05, 2023. doi:10.1001/jamanetworkopen.2022.49591

Data
Data available: No
